# Shapeshifting fungi: How morphological transitions can influence pathogenesis

**DOI:** 10.1371/journal.ppat.1014035

**Published:** 2026-03-13

**Authors:** Gabrielle Paniccia, Carolyn Elya

**Affiliations:** Department of Molecular and Cellular Biology, Harvard University, Cambridge, Massachusetts, United States of America; University of Maryland, Baltimore, UNITED STATES OF AMERICA

## Introduction

**Fig 1:**
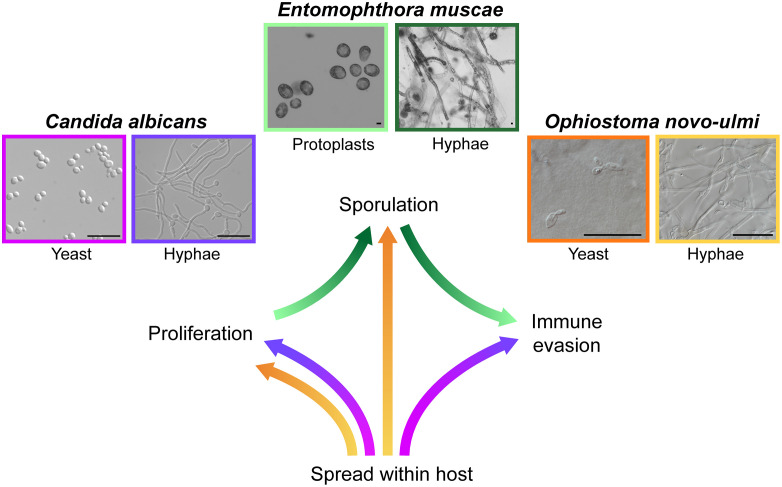
Fungal morphological plasticity enables different pathogenic strategies. (Above) The three species of fungi addressed herein and representative images of their morphologies at key stages. (Below) Schematic of morphological transitions made by these fungi and their corresponding roles in pathogenesis. Arrows are colored according to morphological transitions, corresponding to borders of representative images: color at the base of the arrow indicates the initial morphology, and the color at the tip of the arrow is the transitioned morphology. Scale bars each 20 microns. *Candida albicans* images credit Dr. Teresa O’Meara; *E. muscae* images credit Dr. Gabrielle Paniccia; *O. novo-ulmi* images credit Dr. Tuan Duong.

Fungal pathogens pose an increasing global threat to humans [1], animals [2], and crops [3]. A hallmark of many successful fungal pathogens is their ability to change morphology during infection. Transitions between life-cycle stages—such as forming robust spores, dispersible yeasts, or invasive hyphae—enable fungi to invade, spread, and persist in hostile host environments. Elucidating how and why fungi make these transitions is essential for understanding fungal virulence and developing new antifungal strategies. Here, we highlight the strategies of three fungal “shapeshifters” from distinct phyla and hosts (Fig 1) to illustrate how morphological plasticity underlies pathogenesis.

## Shifting to evade: *Entomophthora muscae*

*Entomophthora muscae* is an obligate fungal pathogen of flies best known for its ability to produce a striking “zombie” phenotype that ensures efficient spore dispersal [4,5]. Yet beyond its behavioral manipulation, the lifecycle of *E. muscae* is remarkable for its sequence of unusual morphological transitions and immune-evasive strategies.

Infection begins when a conidium lands on the cuticle of a susceptible fly. The spore germinates, producing a chitinous germ tube that breaches the cuticle. Once through this barrier, *E. muscae* deposits a cell into the hemolymph that differs fundamentally from typical fungal forms: a cell wall-less protoplast. This unique morphology has long been hypothesized to be central to the pathogen’s success [6]. Unlike vertebrates, insects rely exclusively on innate immunity and the recognition of pathogen-associated molecular patterns (PAMPs) to recognize pathogenic threats and mount defensive responses, including the production of antimicrobial peptides (AMPs) [7]. Because insects recognize fungal pathogens primarily through conserved cell wall components like chitin and β-glucans, the absence of a wall would effectively conceal *E. muscae* from immune detection, allowing it to operate largely undetected within the host.

This wall-less lifestyle is common among fungi in the family Entomophthoraceae. Studies with related fungi show that protoplasts are poor inducers of host immune responses [8,9]. Supporting this, *Drosophila* injected with *E. muscae* protoplasts failed to activate the Toll pathway, and mortality was similar between wild-type and immune-compromised flies [10]. When infection occurred through the natural route (spore germination through cuticle) flies lacking Toll or melanization pathways succumbed faster than wild-type, though all still eventually died. These results suggest that immune signaling is triggered briefly during cuticular penetration, when the chitin-rich germ tube exposes fungal PAMPs, but once inside, the unwalled protoplast operates with near immunological impunity.

Upon entering the fly hemolymph, *E. muscae* protoplasts selectively target the fat body for degradation, a major metabolic and immune organ analogous to a combination of mammalian liver and adipose tissue [11]. Fungal cells then proliferate rapidly until the fly’s abdomen appears visibly opaque. As infection nears completion, the fungus undergoes a dramatic morphological shift back to a walled form (hyphae) in preparation for emergence and sporulation. This transition is likely driven by a metabolic cue, potentially linked to exhaustion of fat body resources, though the precise trigger remains unclear. At this stage, fungal cells degrade remaining organs, harvesting the last available nutrients while inducing host behaviors that position the moribund fly for optimal spore dispersal. By the time the fungus reforms its antigenic cell walls, the fat body is destroyed, and the host’s immune capacity is effectively gone. Specialized hyphae then breach the cuticle, and conidiophores erupt through intersegmental membranes to release infectious conidia into the environment, completing the cycle.

Thus, where most fungi rely on a rigid wall for protection, *E. muscae*’s protoplasts achieve the same end by evasion rather than defense. By toggling between hidden and coated forms, the fungus balances stealth within the host and resilience outside it.

## Shifting to invade: *Candida albicans*

*Candida albicans* is a common human gut commensal [12] that can opportunistically cause localized and systemic infections [13,14]. Consequently, the World Health Organization designates *C. albicans* as a fungal pathogen of critical priority [15]. Virulence in systemic *C. albicans* infection relies on shifting between unicellular yeast and filamentous hyphae or pseudohyphae; mutants that cannot switch between yeast and hyphal forms (and vice versa) show attenuated virulence [16,17]. Indeed, yeast-hyphal dimorphism is a recurring theme among many significant mammalian fungal pathogens [18].

While the yeast form of *C. albicans* is typically associated with commensalism, this morphology also plays an important role in fungal dissemination, especially when dispersing from biofilms [19]. In hospital settings, *C. albicans* frequently forms heterogeneous biofilms composed of yeast and filamentous cells on the surfaces of medical devices. When implanted into patients, the yeast cells pass easily from the biofilm to the patient’s bloodstream and, from there, into multiple organs. Attachment to host cells in these organs stimulates the formation of hyphae, which then invade host tissue either by exerting physical force [20] or by inducing host endocytosis through receptor activation and cytoskeletal rearrangement [21].

Shapeshifting of *C. albicans* also facilitates immune escape. When yeast-stage cells are engulfed by macrophages, they can induce pyroptosis in the engulfing macrophage to kill it [22], then become hyphal to break out of the dead cell [23–25]. Notably, these changes in cell morphology also alter the interactions of *C. albicans* with the host immune system. Host dendritic cells activate different populations of T helper cells in response to *C. albicans* yeast and hyphae [23], and the larger hyphae stimulate neutrophils to release extracellular traps that are critical for the host’s ability to clear large pathogens [24].

The importance of shape-shifting to the pathogenesis of *C. albicans* has made fungal morphogenesis an attractive target for the development of new drugs and therapies [25]. Several studies have identified small molecules that inhibit the ability of *C. albicans* to transition from yeast to hyphae [26–28], and antisense oligonucleotides targeting cell wall genes have been effective in treating a mouse model of systemic candidiasis [29]. This new generation of drugs may turn the reliance of *C. albicans* on shapeshifting into a weakness rather than a strength.

## Shifting to spread: *Ophiostoma novo-ulmi*

*Ophiostoma novo-ulmi* is the aggressive causative agent of the current Dutch Elm Disease (DED) pandemic [30]. Its virulence depends on the transition from yeast-like budding spores to hyphae [31]. *O. novo-ulmi* is vectored to host trees by bark beetles (genera *Scolytus* or *Hylurgopinus*) carrying its yeast-like spores. The beetle transmits the fungus into the elm’s xylem—the water-distributing vascular system of the plant—allowing spores to disseminate. Both yeast and hyphal forms grow in the xylem, but it is the hyphal form that enables the fungus to spread between different xyloid veins, breaking through pit membranes to invade adjacent vessels. In this way, the yeast contributes to the vertical spread of the fungus through the tree, while the hyphal form allows for its lateral spread, a strategy similar to the one employed by *C. albicans* in humans. Following host mortality—which can occur within a year of infection—the fungus transitions to saprophytic growth and produces spores that stick to eclosing beetles.

Morphology of *O. novo-ulmi* is dictated by fungal strain, available nutrients, and the inoculum size [32,33]. Nitrogen source, for example, can bias development, with the fungus producing a higher proportion of mycelia if the nitrogen source is ammonium sulfate but more yeast if the source is proline [32]. Salicylic acid, a hormone involved in mediating plant immunity, also stimulates yeast cell production [33]. *O. novo-ulmi* also has quorum-sensing abilities that impact its morphology; when cultures are inoculated with large numbers of yeast-like spores (10^8^ spores/mL), they tend to mostly favor spore over hyphal production [33]. In all of these growth experiments, different strains of *O. novo-ulmi* produced slightly different responses, with some strains showing stronger commitment to producing one fungal morphology than the other, even with stimulation.

Although the molecular pathogenesis of *O. novo-ulmi* is still being explored, transcriptomic studies have identified candidate genes involved in the yeast-to-hyphal transition [34] as well as candidate virulence genes [35]. Some among this latter category include genes that encode production of toxins, though these will need to be further investigated to assess their contribution to pathogenicity [36]. Future research into antagonists of *O. novo-ulmi* morphological transition or virulence may yield advances that turn the tide in the ongoing DED pandemic.

## Conclusion

Across phyla and hosts, morphological plasticity serves as a common strategy for overcoming host defenses and optimizing growth. Whether toggling between yeast and hyphae to invade tissues, as seen in *C. albicans* and *O. novo-ulmi*, or shedding the cell wall for concealment like *E. muscae*, structural transitions are crucial in shaping the pathogenesis of these fungi.

While efforts to block morphogenesis in *C. albicans* are underway, the broader importance of fungal “shapeshifting” remains underappreciated. Morphological plasticity is a recurring strategy that underlies the virulence of diverse fungal pathogens. Indeed, while the three examples we highlighted exploit yeast-hyphal transitions, there are other morphological transitions that fungi leverage for pathogenesis. *Cryptococcus neoformans*, for example, forms Titan cells [37]—massive, multinucleate fungal cells that bud to produce normal sized daughters—while *Coccidioides* species form large, endospore-filled spherules [38]. Understanding how pathogenic fungi regulate diverse morphological transitions could have far-reaching implications for developing effective antifungal treatments across phyla.
